# Coffee Consumption and Prostate Cancer Risk: Results from National Health and Nutrition Examination Survey 1999–2010 and Mendelian Randomization Analyses

**DOI:** 10.3390/nu13072317

**Published:** 2021-07-05

**Authors:** Menghua Wang, Zhongyu Jian, Chi Yuan, Xi Jin, Hong Li, Kunjie Wang

**Affiliations:** 1 Department of Urology, Institute of Urology (Laboratory of Reconstructive Urology), West China Hospital, Sichuan University, Chengdu 610041, China; 2016181622017@stu.scu.edu.cn (M.W.); jzyhx@scu.edu.cn (Z.J.); 2015141482225@stu.scu.edu.cn (C.Y.); jinxi@scu.edu.cn (X.J.); lihonghxhx@scu.edu.cn (H.L.); 2 West China Biomedical Big Data Center, Sichuan University, Chengdu 610041, China

**Keywords:** coffee, prostate cancer, National Health and Nutrition Examination Survey, Mendelian randomization, PRACTICAL

## Abstract

The aim of this study was to examine the association between coffee and prostate cancer. Firstly, we conducted an observational study using data from National Health and Nutrition Examination Survey (NHANES) 1999–2010. Coffee intake was derived from 24 h dietary recalls. Weighted multivariable-adjusted logistic regression was applied to evaluate the association. Then, we performed Mendelian randomization (MR) to explore the possible causal effect of coffee on prostate cancer risk. Primary and secondary genetic instruments were obtained from genome-wide association studies among 375,833 and 91,462 individuals separately. Prostate cancer summary statistics were extracted from Prostate Cancer Association Group to Investigate Cancer-Associated Alterations in the Genome (PRACTICAL) (79,194 cases and 61,112 controls) and FinnGen project (4754 cases and 63,465 controls). Inverse variance weighted (IVW) was the primary analytical method. Through selection, we enrolled 8336 individuals (weighted number = 58,796,070) for our observational study in NHANES. Results suggested that there was no association between coffee and prostate cancer. MR analyses with primary genetic instruments also did not support a causal association between coffee intake and prostate cancer risk, whether using summary data from PRACTICAL (IVW: OR 1.001, 95% CI 0.997–1.005) or FinnGen (IVW: OR 1.005, 95% CI 0.998–1.012). Similar results were observed when using secondary genetic instruments. Therefore, our study did not support a causal association between coffee intake and prostate cancer risk. Further studies with a larger sample size are needed to examine if an association exists by different coffee bean types, roasting procedures, and brewing methods.

## 1. Introduction

Prostate cancer is the most common solid-organ tumor among men in the developed countries and the second most common worldwide [1]. It is estimated that there will be 248,530 new prostate cancer cases, and 34,130 will die from it in 2021 in the United States (US) [2]. The etiology of prostate cancer is complex, including genetic polymorphisms, positive family history, increased body mass index (BMI), old age, black race, and smoking [3]. In addition to these, recent studies point out that various diet and nutrition factors might be involved in the progression or suppression of prostate cancer [4], among which coffee has aroused researchers’ interest.

Coffee is one of the most commonly consumed beverages worldwide, and its trade exceeded 10 billion dollars a few years ago [5]. Coffee contains hundreds of biologically active substances related to multiple health outcomes like all-cause mortality, cardiovascular disease, and cancer [6]. Several observational studies pointed out that coffee intake might reduce prostate cancer risk [7,8]. Still, the major limitations of these studies were that they were restricted to a particular cohort, and their results might not be representative enough. Moreover, conventional observational studies were susceptible to biases like reverse causation and residual confounding [9].

National Health and Nutrition Examination Survey (NHANES) is a nationally representative survey of American civilians, which provides comprehensive data on various aspects of health and nutrition. NHANES has been used globally by researchers and federal agencies and is considered a remarkable cornerstone for nutrition monitoring in the US [10]. Therefore, NHANES has provided a high-quality and nationally representative sample to explore the correlation between coffee and prostate cancer risk.

Mendelian randomization (MR) is an epidemiological method, which uses genetic variants to evaluate the effect of exposure (e.g., coffee consumption) on an outcome (e.g., prostate cancer risk) [11]. Since the genetic variants are assorted randomly during gamete formation and mostly independent from environmental or lifestyle factors, MR is less vulnerable to the biases from reverse causation and confounding. All these features make MR considered comparable to the randomized controlled trial [12]. One MR study reported no causal effect of coffee intake on prostate cancer risk. However, the inclusion of only two single-nucleotide polymorphisms (SNPs) is a limitation [13].

Therefore, in the current research, we combined an observational study using the nationally representative NHANES and two-sample MR analyses to explore whether there is a causal relationship between coffee and prostate cancer.

## 2. Materials and Methods

### 2.1. Study Population in NHANES

NHANES is a two-year cycle cross-sectional survey in the US [10]. In the present study, we used six consecutive NHANES two-year cycles (1999–2010) in total since participants responded to the questions of “Have you ever been told by a doctor or health professional that you have prostate cancer?” in these six cycles. Among all the participants, we excluded the following individuals: (1) female participants; (2) individuals with missing information about prostate cancer; (3) individuals with incomplete data about dietary intake, BMI, and education level; (4) individuals with excessive energy intake (±three standard deviation energy).

### 2.2. Coffee Consumption Assessment in NHANES

There was only one 24-h dietary recall for individuals in the 1999–2000 and 2001–2002 cycles, while the other four cycles included two dietary recalls. For those with two 24-h dietary recalls, we would use the average coffee consumption from two 24-h recalls. We identified all the coffee beverages in NHANES according to the US Department of Agriculture Dietary Source of Nutrients Database [14].

According to the previous study, we defined one cup size of coffee as 283.5 g in our research [15], and we further divided coffee intake into five groups: no consumption, <1, 1−<2, 2−<4, and 4+ cups/day.

### 2.3. Covariates Used in NHANES

According to the previous study, we used the following variables as covariates: age, BMI, smoking status, education level, and diabetes condition, and intake of energy, alcohol, and calcium [16]. In addition to these, through screening relevant research, history of hypertension, and intake of fat, protein, carbohydrate, polyunsaturated fatty acids, cholesterol, magnesium, and Vitamins A and E might also be associated with prostate cancer risk [4,17] and were therefore included as covariates.

### 2.4. Genetic Instruments for Coffee Consumption

Our primary genetic instruments for coffee consumption were derived from the most recent genome-wide association study (GWAS) among over 370,000 adults of European ancestry, in which they identified 15 significant SNPs associated with self-reported coffee consumption [18]. Secondary genetic instruments were obtained from another genome-wide meta-analysis conducted by the Coffee and Caffeine Genetics Consortium (CCGC), which enrolled 91,462 participants and identified 10 significant SNPs [19]. But in the current study, those SNPs in linkage disequilibrium (r^2^ = 0.01, 10,000 kb) would be excluded. Table S1 showed the list of primary and secondary genetic instruments used in this study.

### 2.5. Genetic Summary Data of Prostate Cancer

We obtained summary data for genetic associations with prostate cancer from the Prostate Cancer Association Group to Investigate Cancer-Associated Alterations in the Genome (PRACTICAL) consortium [20]. Briefly, their study enrolled 79,194 cases and 61,112 controls. To validate our results, we also used the prostate cancer summary data from the FinnGen research project, which had 4754 prostate cancer cases and 63,465 controls (C3_PROSTATE_EXALLC). Researchers can find detailed information about FinnGen on their official website [21].

### 2.6. Statistical Analysis

When performing the observational study in NHANES, we used weighted multivariable-adjusted logistic regression to calculate the odds ratio (OR) with 95% confidence interval (CI) for the prostate cancer risk across coffee intake categories. Due to the multistage and probability cluster design of NHANES, we considered weights in our study. Since we combined six cycles in the present study, we calculated the new weights according to the NHANES tutorials and have placed the detailed formula in the Table S2.

As for MR analyses, we calculated F statistics to evaluate the strength of each instrument. Inverse variance weighted (IVW) was the primary method to assess the association of genetically predicted coffee consumption and prostate cancer risk. We also used MR-Egger, weighted-median, and weighted mode to validate the results from IVW. The advantages and disadvantages of these methods have been discussed in a previously published article [22]. We used Cochrane *Q* test and intercept from MR-Egger to test the possible heterogeneity and directional pleiotropy separately [23]. Leave-one-out sensitivity analysis was also performed.

We used Stata 15.0 (Stata Corporation, College Station, TX, USA) and R (version 3.6.2) to perform all the analyses, and *p* < 0.05 was considered statistically significant.

## 3. Results

### 3.1. Coffee Intake and Prostate Cancer Risk in NHANES

From 1999 to 2010, a total of 62,160 individuals participated in the NHANES. After exclusion, we enrolled 8336 participants (weighted N = 58,796,070) in the final analysis (Figure S1). The median age of included individuals was 54.0 (46.0–64.0), and the weighted incidence of prostate cancer was 2.7% (Table S3).

The baseline characteristics by coffee consumption categories are presented in Table 1. Results from our weighted logistic regression indicated that there was no significant relationship between coffee intake and prostate cancer risk across all the coffee consumption categories (Table 2).

### 3.2. MR Analyses Using Primary Genetic Instruments

**Figure 1:**
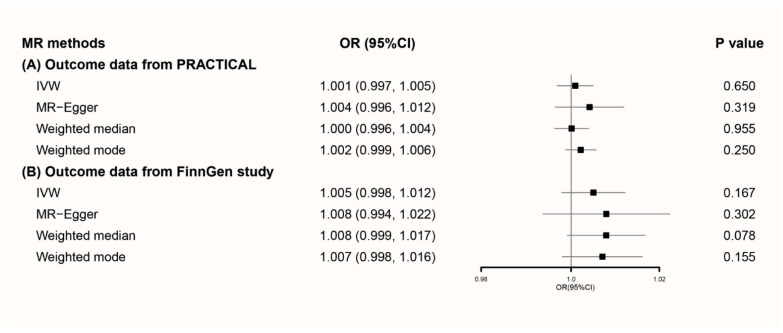
Forest plot of MR using primary genetic instruments with prostate cancer outcome data from (**A**) PRACTICAL and (**B**) FinnGen. MR, Mendelian randomization; OR, odds ratio; CI, confidence interval; IVW, inverse variance weighted; PRACTICAL, Prostate Cancer Association Group to Investigate Cancer-Associated Alterations in the Genome.

Applying primary genetic instruments to PRACTICAL prostate cancer summary statistics, pooled OR for 1% coffee consumption change per allele was 1.001 (95% CI 0.997–1.005) when using IVW. Similar results were observed in MR-Egger, weighted median, and weighted mode methods (Figure 1A). The scatter plot of these results was presented in Figure S2. Potential heterogeneity was detected, but there was no sign of directional pleiotropy (*p* = 0.362) (Table S4). Sensitivity analysis showed that the overall result might be changed when removing rs2330783 and rs1260326 (Figure S3).

Our findings were validated when applying primary genetic instruments to FinnGen prostate cancer summary statistics (Figure 1B). A scatter plot of MR results is presented in Figure S4. There was no sign of heterogeneity (*p* = 0.440 for IVW, *p* = 0.373 for MR-Egger) or directional pleiotropy (*p* = 0.652) (Table S4). Sensitivity analysis showed the overall result would not be changed by removing any SNP (Figure S5).

### 3.3. MR Analyses Using Secondary Genetic Instruments

**Figure 2:**
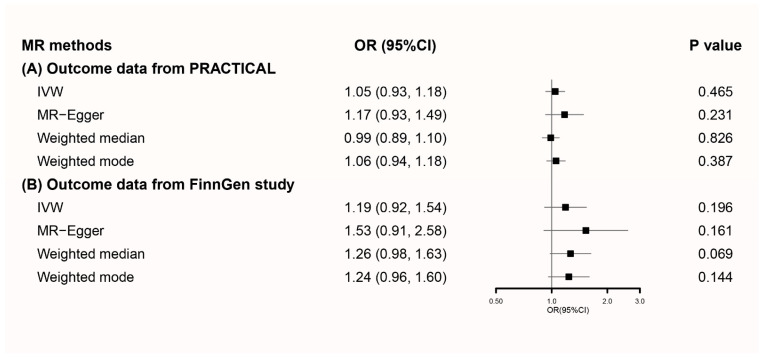
Forest plot of MR using secondary genetic instruments with prostate cancer outcome data from (**A**) PRACTICAL and (**B**) FinnGen. MR, Mendelian randomization; OR, odds ratio; CI, confidence interval; IVW, inverse variance weighted; PRACTICAL, Prostate Cancer Association Group to Investigate Cancer-Associated Alterations in the Genome.

Applying secondary genetic instruments to PRACTICAL prostate cancer summary statistics, pooled OR for cups/day coffee consumption was 1.05 (95% CI 0.93–1.18) when using IVW. Similar results were observed in MR-Egger, weighted median, and weighted mode methods (Figure 2A). A scatter plot of these results is presented in Figure S6. Potential heterogeneity was detected, but there was no sign of directional pleiotropy (*p* = 0.309) (Table S4). Sensitivity analysis showed that the overall result might be changed when removing rs2472297 and rs1260326 (Figure S7).

Findings were validated when applying secondary genetic instruments to FinnGen prostate cancer summary statistics (Figure 2B). The scatter plot of MR results is presented in Figure S8. There was no sign of heterogeneity or directional pleiotropy (Table S4). Sensitivity analysis is presented in Figure S9.

## 4. Discussion

In the current research, we combined an observational study using the nationally representative NHANES 1999–2010 and causal two-sample MR analyses to explore the correlation between coffee intake and prostate cancer risk. Our findings indicate that coffee consumption is unlikely to be causal determinant of prostate cancer.

Coffee is a complex mixture of more than 1000 bioactive ingredients, including caffeine, chlorogenic acids, and various minerals et al. Many of these compounds have been shown to possess anticancer potentials [24]. But whether coffee could reduce the prostate cancer risk was controversial. In a cohort study conducted by Pounis [8], during an average follow-up of 4.24 years, 100 out of 6989 men suffered from prostate cancer. After adjusting cofounders, they found that drinking >3 cups of Italian-style coffee a day could reduce the prostate cancer risk compared to drinking 0–2 cups/day (*p* = 0.02). One advantage of this study was that since the methods of preparing coffee, either using a coffee pot, an Italian coffee pot, or a pot, could influence the compounds in the finally consumed coffee [8], they studied the Italian-style coffee specifically, making the bias from different coffee brewing methods minimal. However, this study’s main disadvantages were the relatively small sample size and limited duration of follow-up, making the power of their observed association limited. Moreover, their results have been questioned by another prospective observational study [16], in which during around 14 years of follow-up, 7036 out of 142,196 men were diagnosed with prostate cancer. And they reported no evidence of association when comparing the highest versus lowest coffee consumption categories (HR 1.02, 95% CI 0.94–1.09), which was consistent with our findings from the nationally representative NHANES. One common weakness of the prospective observational study and our nationally representative NHANES study was that information about the brewing methods was not available.

Still, the major concern with existing conventional observational studies is bias caused by unmeasured or uncontrolled confounding. Previously, many findings from observational studies have been doubted. For example, higher circulating vitamin D used to be correlated with a higher prostate cancer risk according to conventional observational studies (relative risk 1.15, 95% CI 1.06–1.24) [25]. However, in one latest MR, after analyzing genetic data from 79,148 cases and 61,106 controls, researchers pointed out that circulating vitamin D was not causally related to prostate cancer (OR 1.00, 95% CI 0.93–1.07) [26]. For this reason, apart from using the nationally representative observational study in NHANES, MR, which is less vulnerable to biases, has been applied in our study. There is one MR analysis investigating the correlation between coffee and prostate cancer risk already, in which the authors used rs4410790 and rs2472297 as IVs. By analyzing summary data on prostate cancer from around 46,000 men, they found that genetic risk score was not correlated with prostate cancer risk (OR 1.01, 95% CI: 0.98–1.03) [13], which is consistent with our MR findings. However, compared to the MR from Taylor, our analyses have the following advantages. First, in addition to rs4410790 and rs2472297, we used more coffee consumption-associated SNPs in our analyses, making our IVs have a higher explanation of the phenotypic variance. Second, we conducted a total of four different two-sample MR analyses in our study, the results of which were consistent with each other, making our results more robust.

One point that needs to be emphasized is that coffee compounds vary among different coffee species and roasting degree. For instance, it’s reported that *Coffea arabica* contains more lipids, while *Coffea canephora* contains more caffeine polyphenols [27]. Additionally, another study reported that as the degree of roasts increased, the caffeoylquinic acid concentration decreased [28]. These factors might lead to some potential biases. However, according to our knowledge, almost all of the existing research, including ours, did not inquire about the type of coffee beans, roasting degree, and the brewing methods mentioned above. Therefore, for future studies, when designing the food frequency questionnaire to evaluate coffee consumption, it would be useful to add questions about these factors. Further studies with a larger sample size are needed to examine if an association exists by different coffee bean types, roasting procedures, and brewing methods.

The present study has several strengths. First, since diet might play a possible role in prostate cancer’s pathogenesis [4], we included many dietary factors as covariates in our observational study, making our results more reliable. Second, in addition to observational study from the nationally representative NHANES, we also included causal two-sample MR analyses in our study, and the results from the four MR analyses were consistent with each other, making the conclusions of our study more credible and reliable. However, our study could not avoid limitations. First, due to the lack of information on prostate cancer stage, e.g., localized, advanced, or fatal, we did not perform subgroup analysis according to the cancer stages in the present research. In addition, although evidence from our study did not support a causal association between coffee intake and prostate cancer risk, we could not completely rule out the possibility that the effect size might be relatively small, and the sample size of our study was not large enough to detect this difference. Nevertheless, even if such a potential effect was detected when incorporating additional databases, it would be relatively small and was unlikely to have a significant clinical benefit. Last, the type of coffee beans, roasting, and brewing methods were not available in our study, and we included all types of coffee as a whole.

## 5. Conclusions

Our study did not support a causal association between coffee intake and prostate cancer risk. Further studies with a larger sample size are needed to examine if an association exists by different coffee bean types, roasting procedures, and brewing methods.

## Figures and Tables

**Table 1:** Weighted selected characteristics of study sample by coffee consumption categories, NHANES 1999–2010 (Weighted N = 58,796,070).

	Coffee, Cup ^a^/Day				
	0	<1	1−<2	2−<4	4+
Unweighted N	2611	1598	1879	1725	523
Age [years, Median (IQR)]	50 (43−60)	55 (47−67)	57 (48−68)	54 (47−64)	53 (47−61)
Race/ethnicity (%)					
Mexican American	6.0	10.3	6.4	3.7	2.4
Other Hispanic	3.3	7.8	3.9	2.8	0.9
Non-Hispanic White	70.4	64.0	78.5	88.1	93.3
Non-Hispanic Black	16.3	11.1	7.7	3.3	2.5
Other race, including multi-racial	4.0	6.7	3.5	2.1	0.8
Education level (%)					
Less than 9th grade	6.5	12.8	8.1	5.1	5.6
9–11th grade	13.5	12.8	10.4	9.6	13.1
High school graduate/GED or equivalent	25.7	21.2	24.2	24.2	30.1
Some college or AA degree	24.3	27.3	23.7	29.6	28.7
College graduate or above	30.0	25.9	33.6	31.6	22.4
Smoked at least 100 cigarettes in life (%)					
Yes	45.4	60.4	62.0	69.3	80.4
No	54.6	39.6	38.0	30.7	19.6
Overweight/obese (≥25 kg/m^2^) (%)					
Yes	76.2	77.2	75.6	80.0	74.9
No	23.8	22.8	24.4	20.1	25.1
Hypertension (%)					
Yes	33.4	44.9	39.5	35.9	31.0
No	66.6	55.1	60.5	64.1	69.0
Diabetes (%)					
Yes	11.7	13.4	12.6	8.4	9.3
No	86.2	84.6	85.6	89.0	89.5
Borderline	2.1	2.0	1.8	2.5	1.2
Daily intake [Median (IQR)]					
Total energy (kcal)	2317.0 (1823.5−3005.9)	2069.0 (1589.5−2685.5)	2169.0 (1730.5−2722.0)	2328.0 (1798.0−2934.4)	2327.8 (1808.0−2998.0)
Protein (gm)	88.6 (68.4−116.0)	80.0 (61.6−102.3)	84.9 (66.4−108.0)	91.5 (70.4−116.8)	91.1 (69.5−115.9)
Carbohydrate (gm)	286.3 (210.4−368.9)	252.1 (187.6−321.7)	252.4 (195.2−326.7)	258.1 (196.7−332.3)	261.7 (198.3−335.4)
Total fat (gm)	84.8 (59.6−116.5)	76.3 (53.6−104.8)	81.3 (59.9−108.6)	87.8 (62.8−119.1)	95.0 (70.3−124.7)
Total polyunsaturated fatty acids (gm)	17.3 (11.6−25.2)	15.4 (10.7−21.9)	16.9 (11.8−23.6)	17.5 (11.8−25.0)	17.4 (12.8−24.3)
Cholesterol (mg)	271.0 (167.0−410.9)	271.5 (166.0−429.0)	281.0 (177.5−413.0)	304.0 (185.0−457.0)	296.0 (186.5−469.7)
Calcium (mg)	887.1 (581.0−1261.0)	783.0 (544.0−1126.0)	840.0 (574.0−1152.0)	868.5 (617.5−1223.5)	883.0 (630.0−1251.8)
Magnesium (mg)	306.4 (222.5−389.5)	275.0 (214.0−354.0)	305.4 (227.5−392.5)	320.1 (253.0−418.9)	322.0 (260.0−418.0)
Alcohol (gm)	0 (0−7.55)	0 (0−13.0)	0 (0−16.3)	0 (0−24.0)	0 (0−17.4)
Caffeine (mg)	53.5 (2.86−129.0)	98.5 (61.0−156.5)	189.5 (137.0−245.0)	332.0 (256.0−420.0)	678.0 (528.5−860.9)
Vitamin A (mcg)	607.6 (353.5−925.5)	546.5 (333.0−850.0)	590.5 (360.0−884.5)	625.0 (389.0−965.0)	535.0 (345.0−863.0)
Vitamin E (mcg)	7.4 (5.0−10.8)	6.6 (4.5−9.2)	7.4 (5.0−10.3)	7.7 (5.2−10.7)	7.2 (5.2−10.4)

^a^ one cup is defined as 283.5 g of coffee; IQR, interquartile range; NHANES, National Health and Nutrition Examination Survey.

**Table 2:** Weighted multivariable-adjusted ^a^ logistic regression of prostate cancer risk across coffee consumption categories in NHANES 1999–2010 (Weighted N = 58,796,070).

Coffee Consumption (Cup ^b^/Day)	OR	95% CI		*p* Value
		Lower Limit	Upper Limit	
0	Ref	-	-	-
<1	1.18	0.77	1.80	0.439
1−<2	1.42	0.96	2.10	0.078
2−<4	1.53	0.90	2.61	0.114
4+	1.81	0.86	3.79	0.116

^a^ Adjust for age (continuous), race and ethnicity (Mexican American, other Hispanic, non-Hispanic White, non-Hispanic Black, Other race including multi-racial), education level (less than 9th grade, 9–11th grade, high school graduate/GED or equivalent, some college or AA degree, college graduate or above), smoking status (smoked at least 100 cigarettes in life or not), body mass index (continuous), hypertension (yes or no), diabetes (yes, no, or borderline), and dietary intake of total energy, protein, carbohydrate, total fat, total polyunsaturated fatty acids, cholesterol, calcium, magnesium, alcohol, vitamin A and vitamin E. ^b^ One cup is defined as 283.5 g of coffee. NHANES, National Health and Nutrition Examination Survey; OR, odds ratio; CI, confidence interval.

## Data Availability

The data used in this study were publicly available and can be accessed via the references in the manuscript.

## Supplementary Materials

The following are available online at https://www.mdpi.com/article/10.3390/nu13072317/s1, Figure S1: Flow chart of eligible participant selection for the nationally representative observational study in NHANES 1999–2010, Figure S2: Scatter plot of the genetic association with coffee consumption against genetic association with prostate cancer using primary genetic instruments and PRACTICAL Consortium prostate cancer summary statistics, Figure S3: Leave-one-out sensitivity analysis for the MR using primary genetic instruments and PRACTICAL Consortium prostate cancer summary statistics, Figure S4: Scatter plot of the genetic association with coffee consumption against genetic association with prostate cancer using primary genetic instruments and FinnGen prostate cancer summary statistics, Figure S5: Leave-one-out sensitivity analysis for the MR using primary genetic instruments and FinnGen prostate cancer summary statistics, Figure S6: Scatter plot of the genetic association with coffee consumption against genetic association with prostate cancer using secondary genetic instruments and PRACTICAL prostate cancer summary statistics, Figure S7: Leave-one-out sensitivity analysis for the MR using secondary genetic instruments and PRACTICAL Consortium prostate cancer summary statistics, Figure S8: Scatter plot of the genetic association with coffee consumption against genetic association with prostate cancer using secondary genetic instruments and FinnGen prostate cancer summary statistics, Figure S9: Leave-one-out sensitivity analysis for the MR using secondary genetic instruments and FinnGen prostate cancer summary statistics, Table S1: Genetic instruments used in our Mendelian randomization studies, Table S2: Weights used in our NHANES 1999–2010 analysis, Table S3: Baseline characteristics of included individuals in NHANES 1999–2010 (Weighted N = 58,796,070), Table S4: Summary on MR results of coffee consumption on prostate cancer risk.
