# Supplementary material for: Coffee Consumption and Prostate Cancer Risk: Results from National Health and Nutrition Examination Survey 1999–2010 and Mendelian Randomization Analyses
Source: Nutrients. 2021 Jul 5;13(7):2317. doi: 10.3390/nu13072317 (PMC8308488; doi:10.3390/nu13072317)
Supplement: Supplementary file 1 [file nutrients-13-02317-s001.zip › nutrients-1227473-supplementary.pdf]

Supplementary Material

Figure S1. Flow chart of eligible participants selection for the nationally representative observational study in NHANES 1999-2010. NHANES, National Health and Nutrition Examination Survey.

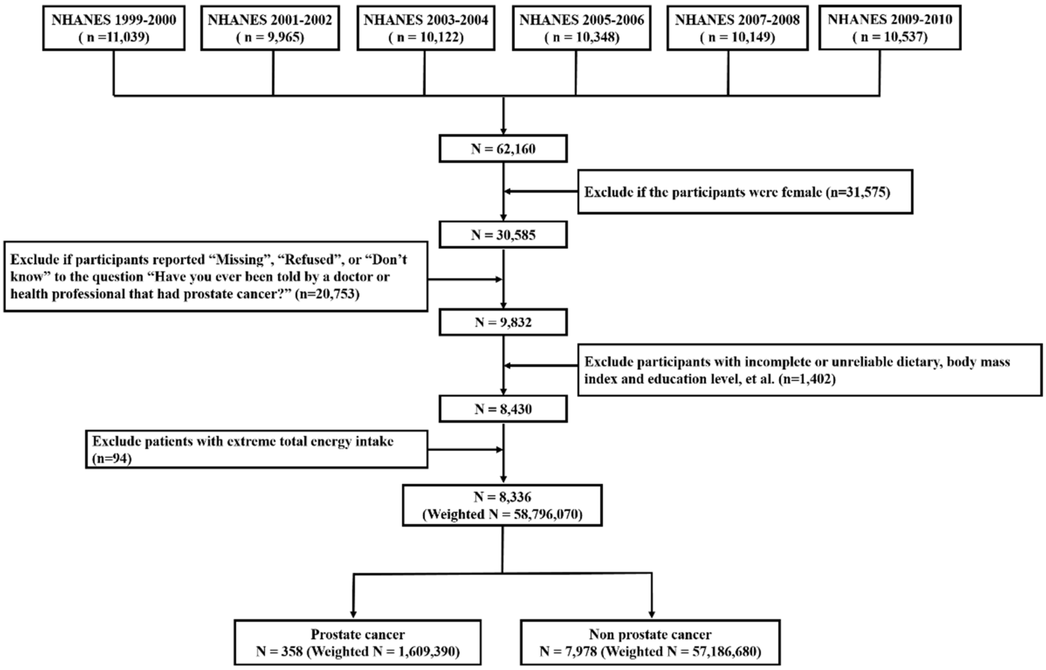

Figure S2. Scatter plot of the genetic association with coffee consumption against genetic association with prostate cancer using primary genetic instruments and PRACTICAL Consortium prostate cancer summary statistics. SNP, single nucleotide polymorphism; PRACTICAL, Prostate Cancer Association Group to Investigate Caner-Associated Alterations in the Genome

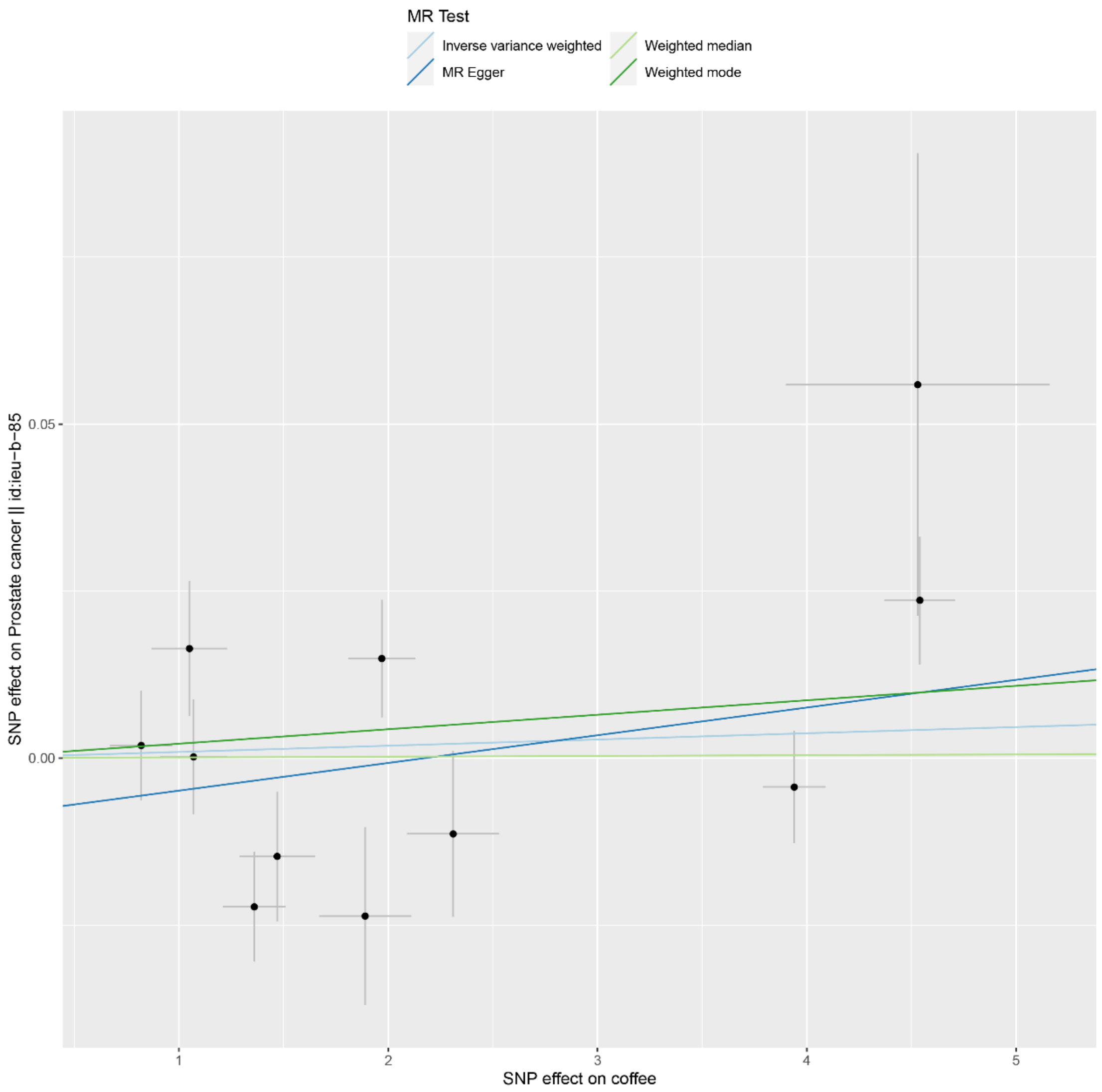

Figure S3. Leave-one-out sensitivity analysis for the MR using primary genetic instruments and PRACTICAL Consortium prostate cancer summary statistics. MR, Mendelian randomization; PRACTICAL, Prostate Cancer Association Group to Investigate Caner-Associated Alterations in the Genome; IVW, Inverse variance weighted

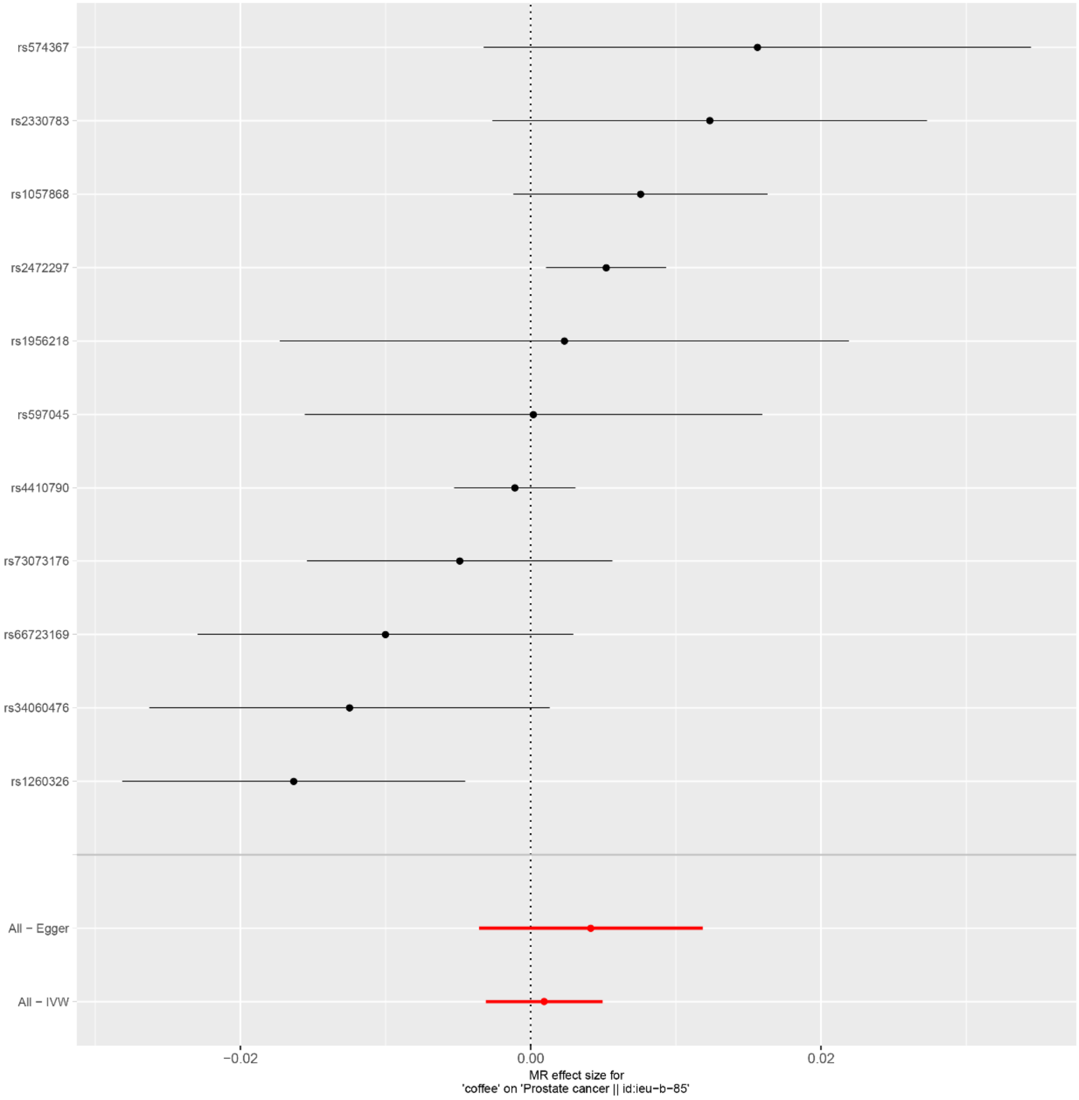

Figure S4. Scatter plot of the genetic association with coffee consumption against genetic association with prostate cancer using primary genetic instruments and FinnGen prostate cancer summary statistics. SNP, single nucleotide polymorphism

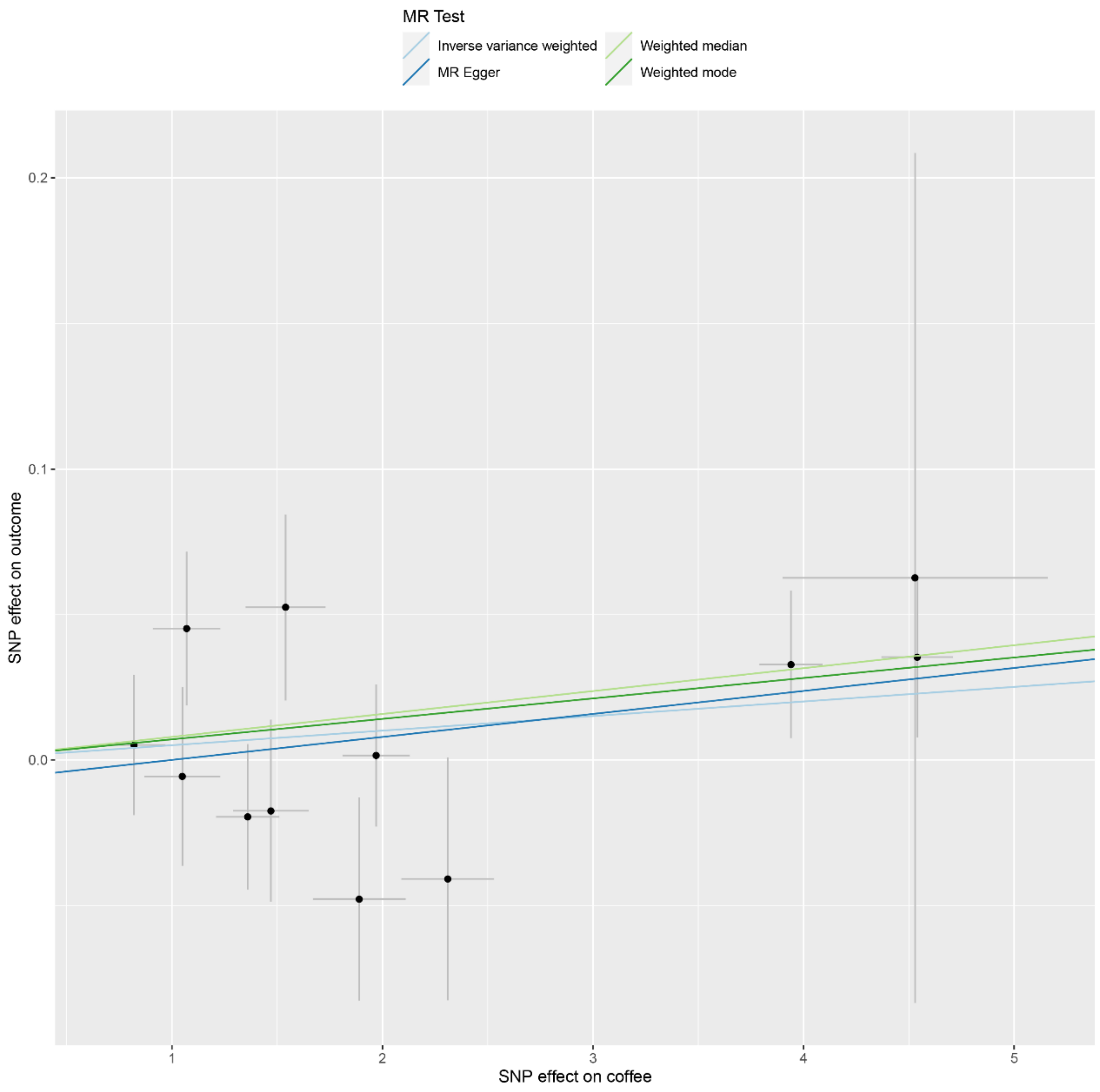

Figure S5. Leave-one-out sensitivity analysis for the MR using primary genetic instruments and FinnGen prostate cancer summary statistics. MR, Mendelian randomization; IVW, Inverse variance weighted

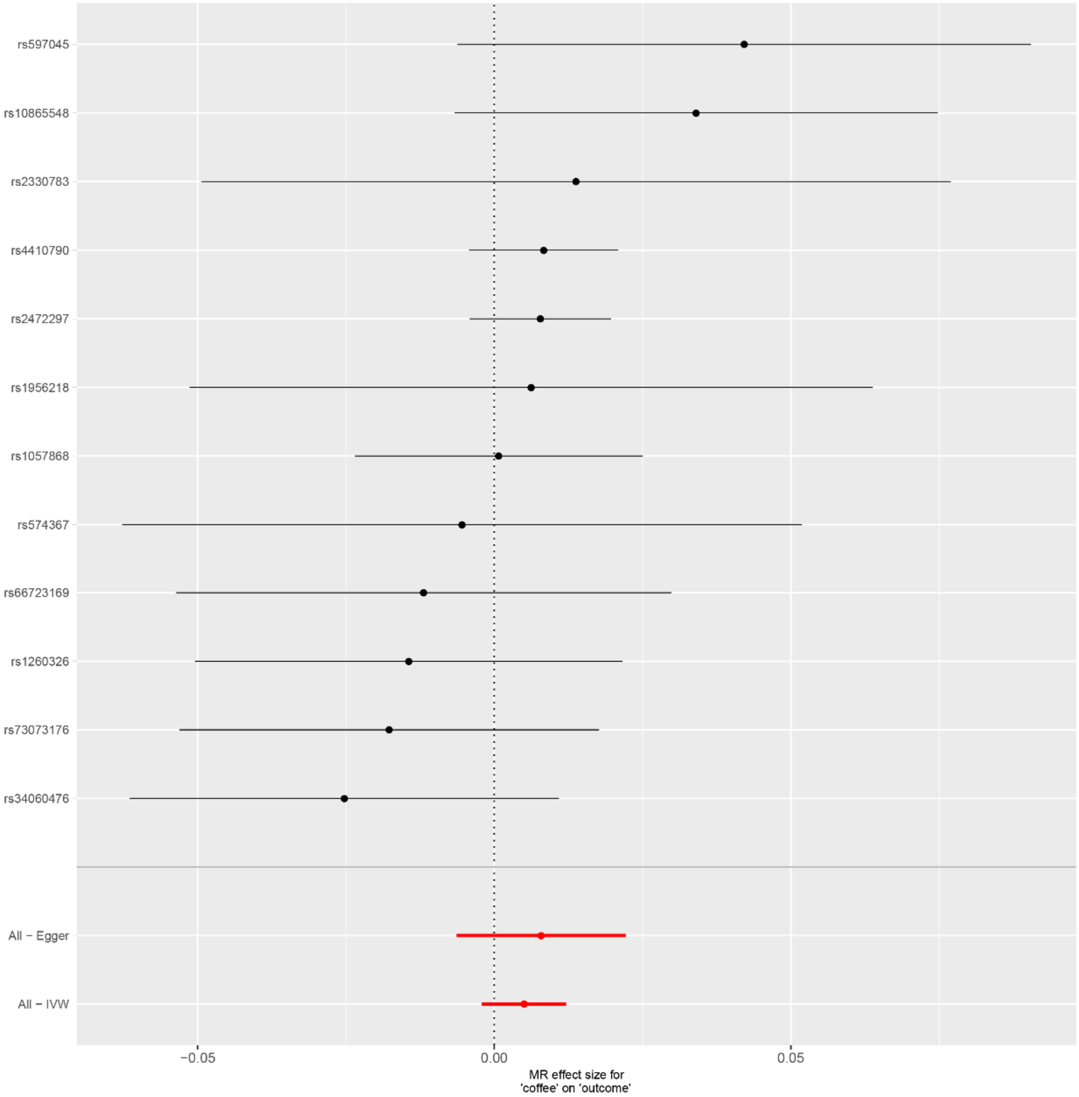

Figure S6. Scatter plot of the genetic association with coffee consumption against genetic association with prostate cancer using secondary genetic instruments and PRACTICAL prostate cancer summary statistics. SNP, single nucleotide polymorphism; PRACTICAL, Prostate Cancer Association Group to Investigate Caner-Associated Alterations in the Genome

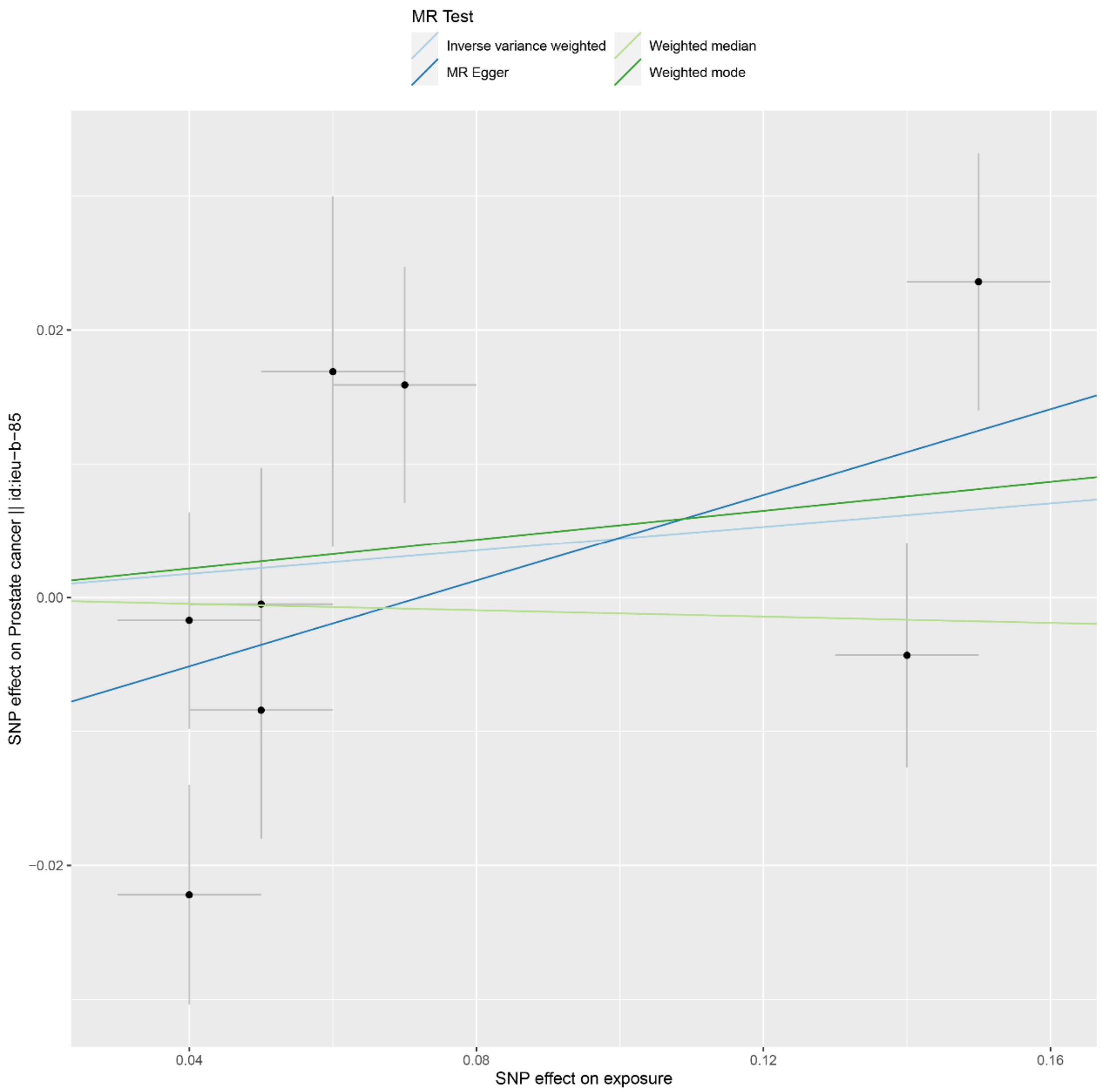

Figure S7. Leave-one-out sensitivity analysis for the MR using secondary genetic instruments and PRACTICAL Consortium prostate cancer summary statistics. MR, Mendelian randomization; PRACTICAL, Prostate Cancer Association Group to Investigate Caner-Associated Alterations in the Genome; IVW, Inverse variance weighted

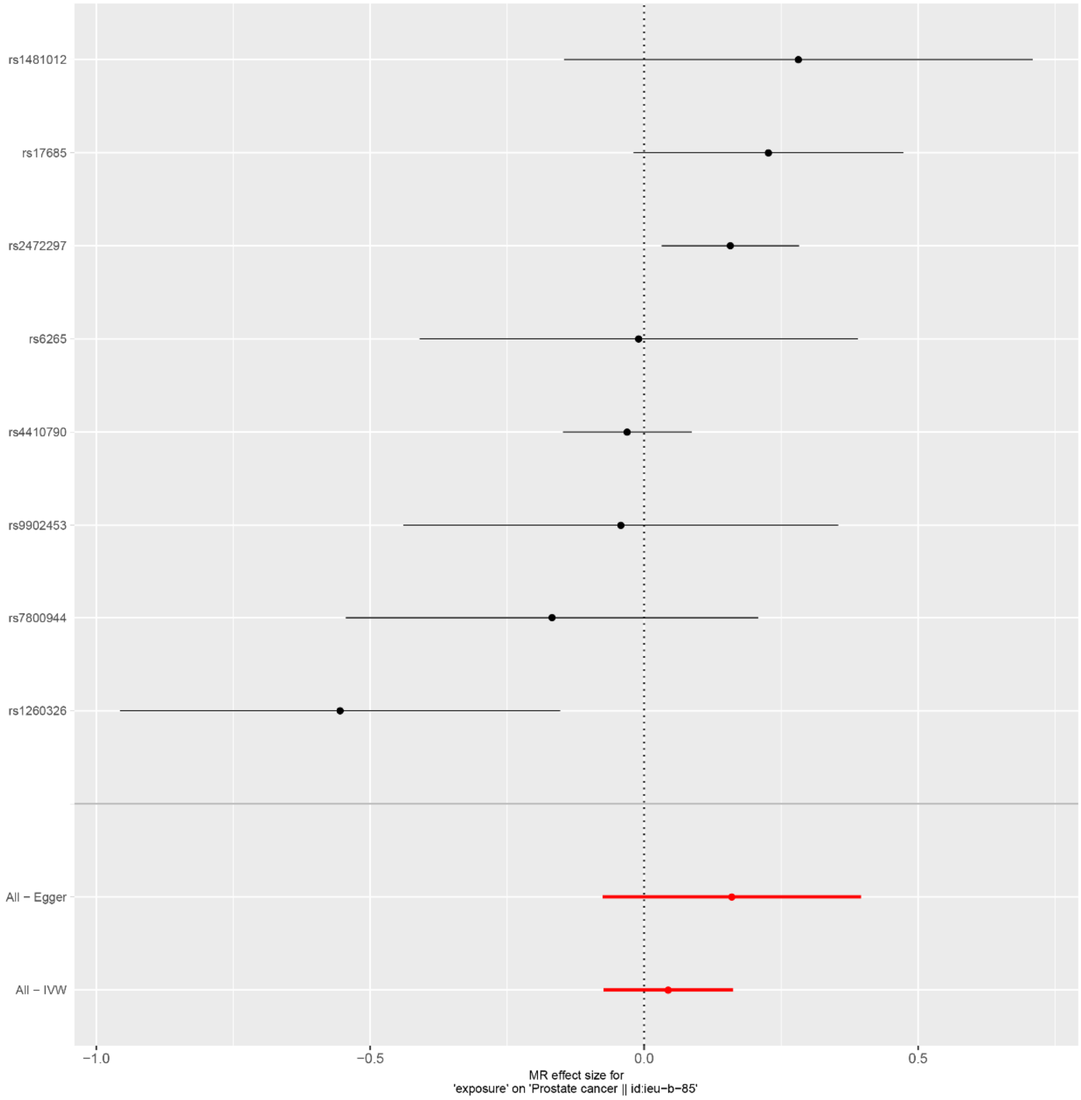

Figure S8. Scatter plot of the genetic association with coffee consumption against genetic association with prostate cancer using secondary genetic instruments and FinnGen prostate cancer summary statistics. SNP, single nucleotide polymorphism

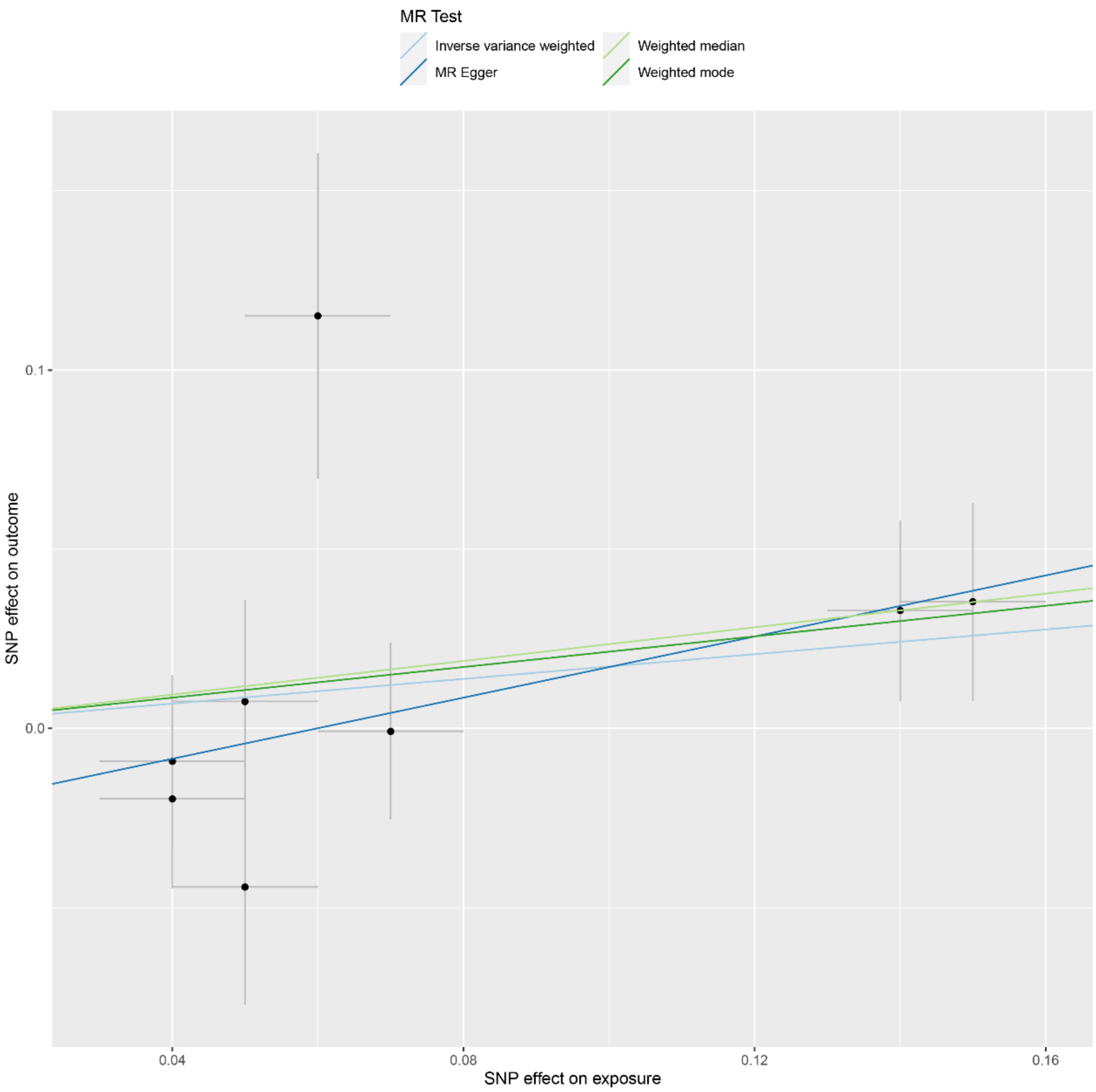

Figure S9. Leave-one-out sensitivity analysis for the MR using secondary genetic instruments and FinnGen prostate cancer summary statistics. MR, Mendelian randomization; IVW, Inverse variance weighted

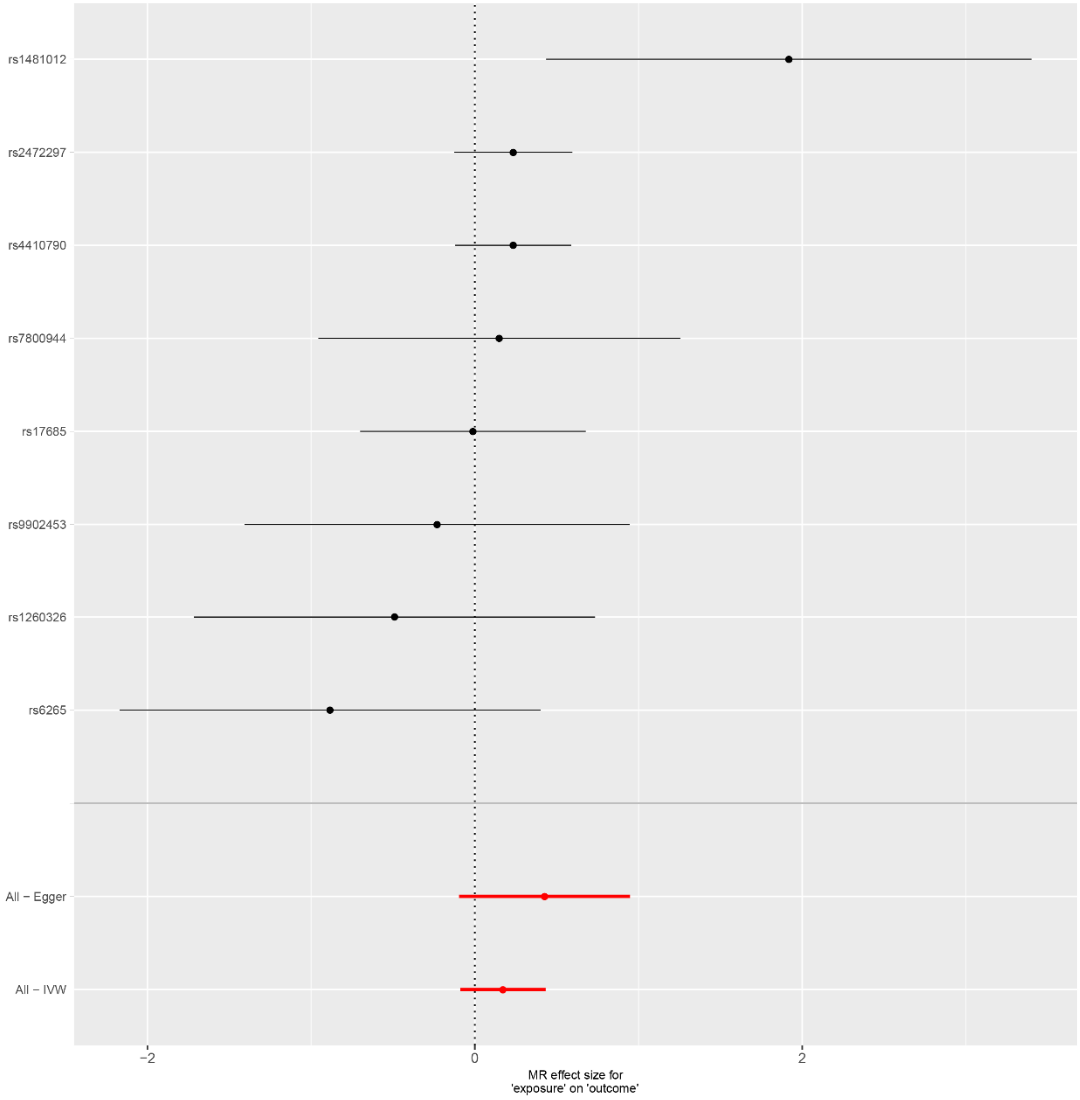

Table S1. Genetic instruments used in our Mendelian randomization studies

SNPs	Nearest gene	EA	EAF	Variance explained (r^2)	F statistic	Exposure		PRACTICAL outcome		FinnGen outcome		Genetic instruments
						β	SE	β	SE	β	SE	
rs1057868	POR	T	0.29	3.81E-04	143	1.97	0.16	0.0149	0.0088	0.0015	0.0244	Primary genetic instruments
rs10865548	TMEM18	G	0.83	1.64E-04	62	1.54	0.19	NA[#]	NA[#]	0.0524	0.032	
rs1260326	GCKR	C	0.61	2.15E-04	81	1.36	0.15	-0.0222	0.0082	-0.0196	0.025	
rs1956218	AKAP6	G	0.56	8.07E-05	30	0.82	0.15	0.0019	0.0082	0.0051	0.0241	
rs2330783	SPECC1L-ADORA2A	G	0.99	1.33E-04	50	4.53	0.63	0.0559	0.0346	0.0625	0.146	
rs2472297	CYP1A1/2	T	0.27	1.87E-03	704	4.54	0.17	0.0236	0.0096	0.0353	0.0276	
rs34060476	MLXIPL	G	0.13	1.99E-04	75	1.89	0.22	-0.0236	0.0133	-0.0478	0.0349	
rs4410790	AHR	C	0.63	1.70E-03	640	3.94	0.15	-0.0043	0.0084	0.0328	0.0253	
rs574367	SEC16B	T	0.21	8.85E-05	33	1.05	0.18	0.0164	0.0101	-0.0057	0.0307	
rs597045	OR8U8	A	0.69	1.13E-04	42	1.07	0.16	2.00E-04	0.0086	0.0451	0.0264	
rs66723169	MC4R	A	0.23	1.84E-04	69	1.47	0.18	-0.0147	0.0097	-0.0175	0.0313	
rs73073176	LOC101927630	C	0.87	2.83E-04	106	2.31	0.22	-0.0113	0.0124	-0.0409	0.0417	
rs1260326	GCKR	T	0.41	0.00031	28	-0.04	0.0082	0.0222	0.0082	0.0196	0.025	Secondary genetic instruments
rs1481012	ABCG2	A	0.89	0.00026	24	0.06	0.0131	0.0169	0.0131	0.1151	0.0454	
rs17685	POR	A	0.29	0.00061	56	0.07	0.0088	0.0159	0.0088	-8.00E-04	0.0246	
rs2472297	CYP1A2	T	0.24	0.00226	207	0.15	0.0096	0.0236	0.0096	0.0353	0.0276	
rs4410790	AHR	T	0.37	0.00279	256	-0.14	0.0084	0.0043	0.0084	-0.0328	0.0253	
rs6265	BDNF	T	0.19	0.00028	26	-0.05	0.0102	5.00E-04	0.0102	0.0442	0.0328	
rs7800944	MLXIPL	T	0.72	0.00036	33	-0.05	0.0096	0.0084	0.0096	-0.0075	0.0282	

SNP, single-nucleotide polymorphism; EA, effect allele; EAF, effect allele frequency; PRACTICAL, Prostate Cancer Association Group to Investigate Caner-Associated Alterations in the Genome; SE, standard error; NA, not available

[#] SNP rs10865548 was not found in the PRACTICAL prostate cancer outcome.

Table S2. Weights used in our NHANES 1999-2010 analysis

	NHANES 1999-2002	NHANES 2003-2010
Formula	2/6 * dietary day one 4-Year sample weigh	1/6 * dietary two day sample weight
Stata code	2/6 * wtdr4yr	1/6 * wtdr2d

NHANES, National Health and Nutrition Examination Survey

Table S3. Baseline characteristics of included individuals in NHANES 1999-2010 (Weighted N = 58,796,070)

	Unweighted N	Weighted proportion (%)
Prostate cancer		
Yes	358	2.7
No	7,978	97.3
Race/ethnicity		
Mexican American	1,531	5.9
Other Hispanic	466	3.8
Non-Hispanic White	4,593	77.2
Non-Hispanic Black	1,524	9.4
Other Race-Including Multi-Racial	222	3.6
Education level		
Less than 9th grade	1,383	7.4
9-11th grade	1,286	11.8
High school graduate/GED or equivalent	1,909	24.7
Some college or AA degree	1,941	26.3
College graduate or above	1,817	29.8
Smoked at least 100 cigarettes in life		
Yes	5196	59.8
No	3140	40.2
Overweight/obese (≥25 kg/m2)		
Yes	6,307	77.1
No	2,029	22.9
Hypertension		
Yes	3,473	36.9
No	4,863	63.2
Diabetes		
Yes	1,242	11.1
No	6,906	86.8
Borderline	188	2.1

NHANES, National Health and Nutrition Examination Survey

Table S4. Summary on MR results of coffee consumption on prostate cancer risk

**Genetic instruments**	**OUTCOME**	**MR RESULTS**					**HETEROGENEITY**		**PLEIOTROPY**	
		**Methods**	**N SNPs**	**OR**	**95%CI**	***P* value**	**METHODS**	***P* value**	**METHODS**	***P* value**
Primary genetic instruments	PRACTICAL	IVW	11	1.001	0.997-1.005	0.650	IVW	0.002		
		MR-Egger	11	1.004	0.996-1.012	0.319	MR-Egger	0.003	MR-Egger intercept	0.362
		Weighted median	11	1.000	0.996-1.004	0.955				
		Weighted mode	11	1.002	0.999-1.006	0.250				
Primary genetic instruments	FinnGen	IVW	12	1.005	0.998-1.012	0.167	IVW	0.440		
		MR-Egger	12	1.008	0.994-1.022	0.302	MR-Egger	0.373	MR-Egger intercept	0.652
		Weighted median	12	1.008	0.999-1.017	0.078				
		Weighted mode	12	1.007	0.998-1.016	0.155				
Secondary genetic instruments	PRACTICAL	IVW	8	1.045	0.928-1.176	0.465	IVW	0.012		
		MR-Egger	8	1.174	0.927-1.486	0.231	MR-Egger	0.021	MR-Egger intercept	0.309
		Weighted median	8	0.988	0.889-1.098	0.826				
		Weighted mode	8	1.056	0.941-1.184	0.387				
Secondary genetic instruments	FinnGen	IVW	8	1.188	0.915-1.541	0.196	IVW	0.189		
		MR-Egger	8	1.531	0.908-2.581	0.161	MR-Egger	0.214	MR-Egger intercept	0.316
		Weighted median	8	1.264	0.981-1.629	0.069				
		Weighted mode	8	1.238	0.960-1.597	0.144				

MR, Mendelian randomization; SNP, single-nucleotide polymorphism; OR, odds ratio; PRACTICAL, Prostate Cancer Association Group to Investigate Caner-Associated Alterations in the Genome; IVW, Inverse variance weighted
